# FDA Experience with Medical Countermeasures under the Animal Rule

**DOI:** 10.1155/2012/507571

**Published:** 2011-09-20

**Authors:** Paul Aebersold

**Affiliations:** Global Regulatory Affairs, Quintiles, Inc., 1801 Rockville Pike, Rockville, MD 20852, USA

## Abstract

The Food and Drug Administration issued a final rule in May 2002 to permit the Agency to approve drugs or license biological products on the basis of animal efficacy studies for use in ameliorating or preventing serious or life-threatening conditions caused by exposure to lethal or permanently disabling toxic biological, chemical, radiological, or nuclear substances. Only two drugs were approved in the first nine years of the “Animal Rule” despite massive investment by the federal government since 2001 to stimulate development of medical countermeasures to biological threats. This article therefore examines the Food and Drug Administration reviews made public after approval of those two drugs and the public discussion at the Agency's Anti-Infective Drugs Advisory Committee of one biological product under development under the Animal Rule. Despite the paucity of approved drugs or licensed biological products as medical countermeasures, several investigational drugs have been placed in the National Strategic Stockpile for use as medical countermeasures, if needed.

## 1. Introduction

There is a risk in the post-September 11, 2001, world of chemical, biological, radiological, or nuclear attack on the United States, either on civilian populations or on military forces. To support the development of medical countermeasures (MCM) against such threats, the government has invested an estimated $54 billion in civilian spending from fiscal year 2001 through fiscal year 2010 [1]. Yet despite this massive investment, the United States lacks the range of MCM listed in the Health and Human Services Public Health Emergency MCM Enterprise Implementation Plan [2].

In 2002, the Food and Drug Administration (FDA) issued what has become known as the “Animal Rule,” intended to expedite the development of new drugs and biologic products as MCM to chemical, biological, radiological, and nuclear threats. The Animal Rule applies only to new drug or biologic products for which definitive human efficacy studies cannot be conducted because it would be unethical to deliberately expose healthy human volunteers to a lethal or permanently disabling toxic biological, chemical, radiological, or nuclear substance. 

Since the Animal Rule was issued, only two drug products for humans have been approved by the FDA on the basis of efficacy studies in animals. This paper summarizes the context in which the Animal Rule came into being, the regulatory provisions of the Animal Rule, and the publically-available information on how FDA has reviewed animal efficacy studies in support of drug or biologic product applications for use in humans.

## 2. The Medical Threats

In September 1980, Iraq invaded Iran in a war that lasted until August 1988. During the war, Iran reported that Iraq used chemical weapons on Iranian soldiers. One of the chemical-warfare instances reported by Iran, at Hoor-ul-Huzwaizeh on March 13, 1984, was verified by an international team of specialists dispatched to Iran by the United Nations Secretary General. The evidence adduced in the report by the UN team lends substantial credence to Iranian allegations of Iraqi chemical warfare on at least six other occasions during the period from February 26 to March 17 of that year [3]. 

A decade later in August 1990, Iraq invaded Kuwait. An international coalition of countries deployed troops to Saudi Arabia in preparation to liberate Kuwait. At that time, Iraq was thought to possess biological as well as chemical weapons. 

In the decade following the ensuing Gulf War, the United Nations passed 16 Security Council resolutions calling for the complete elimination of Iraqi weapons of mass destruction. The United Nations Special Commission (UNSCOM) was established to oversee Iraq's compliance with destruction of chemical, biological, and missile weapons facilities [4]. UNSCOM learned that Iraq had ordered tons of growth media from the British company Oxoid, whereas Iraq's hospital consumption of growth media was only 200 kilograms a year. Rihab Rashid Taha, an Iraqi microbiologist educated in England, admitted to UNSCOM inspectors that she had grown 19,000 liters of botulism and 8,000 liters of anthrax, as well as smaller amounts of other dangerous organisms. She also had conducted research with camel pox virus, which raised fears that Iraq had planned to weaponize smallpox virus.

On September 18, 2001, apparently unrelated to events in Iraq, a letter was mailed from Trenton, New Jersey, to NBC news anchor Tom Brokaw in New York City. The letter contained a brown granular material that turned out to be anthrax spores. The staff member who opened the letter developed cutaneous symptoms around September 29 and saw a doctor on October 1. Another letter containing anthrax was mailed on September 18 to the New York Post, where three employees developed cutaneous anthrax. Although never recovered, three other letters are thought to have been mailed at the same time to ABC News and CBS News in New York and to the National Enquirer at the offices of AMI in Boca Raton, Florida. A photo editor at AMI developed inhalation anthrax and died on October 5 [5].

On October 9, 2001, two more letters were mailed from Trenton to Senate Majority Leader Tom Daschle and Senator Patrick Leahy in Washington, DC. Both senators had expressed concerns about the administration's proposed “anti-terrorism” Patriot Act. These October 9 letters contained anthrax spores in the form of a highly refined, dry white powder. Two workers at the Brentwood postal facility near Washington died of inhalational anthrax on October 22. In all, from the four recovered and three suspected letters, five people died from inhalational anthrax and at least 22 others were infected but recovered from either cutaneous or inhalational anthrax. Thousands of other people thought to have been exposed to anthrax were placed on antibiotic therapy [5].

More than a year later, on February 5, 2003, in remarks to the United Nations Security Council, Secretary of State Colin Powell noted that it had taken less than a teaspoon of dry anthrax to shut down the United States Senate [6].

In 2003, Al Qaeda issued a fatwa authorizing the use of biological, chemical, and nuclear weapons against infidels [7]. In March 2005, the Robb-Silverman Report on Weapons of Mass Destruction documented that Al Qaeda had a major bioweapons effort in Afghanistan as of 2003 [8].

## 3. Impasse at FDA on Medical Countermeasures

During the 1980s, the time of the Iraq/Iran war, the Army was investigating pyridostigmine bromide (PB) as a potential protection against the nerve gas soman. The Army discussed its promising animal results with FDA, which explained to the Army in 1988 that it had no regulatory pathway to approve the drug on the basis of animal studies.

In October 1990, in preparation for the Gulf War to liberate Kuwait, the Department of Defense (DoD) requested of FDA that it establish authority to waive its requirement for informed consent for use of investigational drugs [9]. In less than two months, FDA announced an interim final rule, Informed Consent for Human Drugs and Biologics: Determination that Informed Consent is Not Feasible [10]. One week later, DoD requested waivers for PB and anthrax vaccine; 18 days after the request, FDA granted both waivers [9]. Operation Desert Storm began 11 days after the waivers were granted. Under orders, soldiers in combat took PB as an experimental protection against the nerve gas soman. After Kuwait was liberated, a number of veterans suffered from undiagnosed illnesses which collectively came to be known as “Gulf War Syndrome.” Veteran groups alleged that the investigational drugs had caused these illnesses, and a presidential advisory committee on gulf war veterans' illnesses was established in 1995 [11].

In December 1992, FDA promulgated new regulations to facilitate the development of treatments for acquired immuno-deficiency syndrome (AIDS) based on surrogate markers [12]. A surrogate marker, such as CD-4 white blood cell counts, might respond rapidly to a new antiviral treatment, whereas it could take years to evaluate the effect of the new antiviral drug on long term survival of AIDS patients. These “Accelerated Approval” regulations required that a surrogate marker must be reasonably likely to predict clinical benefit. The Army saw in this new regulation a path forward for licensure of PB as a protection against Soman nerve gas and filed a New Drug Application (NDA) with FDA in May, 1996.

Ten months later in March, 1997, Dr. Paul Leber, director of the Division of Neuropharmacological Drug Products at FDA, disputed the Army's claim that the surrogate marker of their clinical trials was reasonably likely to predict clinical benefit for the intended military use. He argued that the predictive power of the surrogate must be shown in humans before any “reasonable person” could predict clinical benefit. He considered that extrapolation of clinical benefit in humans based on animal experiments was unjustified [13].

Dr. Robert Temple, director of the Office of Drug Evaluation I at FDA, did not agree with what he read as Dr. Leber's essentially absolute conclusion that animal data linking inhibition of blood cholinesterase by PB to protection against nerve agents, together with human evidence of inhibition of blood cholinesterase, could *never* be taken as evidence of effectiveness under regulations for Accelerated Approval of New Drugs for Serious or Life-Threatening Illnesses. In his memorandum of August 1998, Dr. Temple noted: “As I wrote that part of the preamble, I agree with it.” General principles aside, however, he did agree that cholinesterase inhibition had not been shown by the Army to be a reasonable surrogate for survival benefit [13]. 

Dr. Leber replied by memorandum in September 1998, noting that Dr. Temple was incorrect in his assertion that he, Dr. Leber, wrote that animal data could “never” be used as a basis for reaching a conclusion under the Accelerated Approval regulations. Dr. Leber noted that his memorandum spoke to why, from an epistemological and scientific perspective, it would be imprudent to extrapolate from an effect observed in an animal model to a conclusion about the effectiveness of the intervention in humans [13].

## 4. FDA's Response to Chemical, Biological, Radiological and Nuclear Threats

### 4.1. The Animal Rule

In July 1997, FDA requested comments on its proposed rule for Accessibility to New Drugs for Use in Military and Civilian Exigencies When Traditional Human Efficacy Studies Are Not Feasible [14]. Specifically, FDA asked the following questions.

1. Should its rule permitting waiver of informed consent in very limited circumstances involving military exigencies be revoked or amended?
2. This question soon became moot.
3. When, if ever, is it ethical to expose volunteers to toxic substances for testing antidotes?
4. FDA received nine comments. One comment suggested that the developers of these drugs, if they are confident that the drugs are both safe and effective, should offer themselves for final testing of safety and efficacy. DoD strongly opposed testing of toxic substances and also stated that testing of sublethal doses of the toxic substances would be uninformative.
5. What evidence of efficacy, other than from human trials, would be appropriate to demonstrate the safety and efficacy of products that may provide protection against toxic chemical and biological substances?
6. FDA received nine comments, most of which did not mention specific types of information that would be needed for approval. The Public Citizen Litigation Group rejected as illegal, without elaboration, the idea that animal data could serve as the basis of approval of an antidote.

This rulemaking was rendered moot by the* National Defense Authorization Act for Fiscal 1999*, which authorized the president to waive FDA's informed consent requirements in certain military situations. Consequently, in October 1999, FDA revoked its 1990 interim final rule and issued a new interim final rule, which established criteria and standards for the president to apply [15].

Also in October 1999, FDA issued a proposed rule, New Drug and Biological Products; Evidence Needed to Demonstrate Efficacy of New Drugs for Use Against Lethal or Permanently Disabling Toxic Substances When Efficacy Studies in Humans Ethically Cannot Be Conducted [16]. The proposed rule would also apply when field trials after accidental or hostile exposure are not feasible. Human safety studies would be needed to support licensure.

FDA received comments on the proposed rule from only two pharmaceutical companies, one physician affiliated with a university, and the National Institutes of Health. The final rule was issued in May 2002, with the revised title, New Drug and Biological Drug Products; Evidence Needed to Demonstrate Effectiveness of New Drugs When Human Efficacy Studies Are Not Ethical or Feasible [17].

This rule has become known as the Animal Rule. The scope of the rule and the standards for effectiveness are reproduced below from the Code of Federal Regulations, as the author, who formerly worked at FDA, studiously avoids paraphrasing regulations:

TITLE 21–FOOD AND DRUGS

CHAPTER I–FOOD AND DRUG ADMINISTRATION

DEPARTMENT OF HEALTH AND HUMAN SERVICES

SUBCHAPTER D–DRUGS FOR HUMAN USE

PART 314 APPLICATIONS FOR FDA APPROVAL TO MARKET A NEW DRUG

**Subpart I—Approval of New Drugs When Human Efficacy Studies Are Not Ethical or Feasible**

(i) Sec. 314.600 Scope. This subpart applies to certain new drug products that have been studied for their safety and efficacy in ameliorating or preventing serious or life-threatening conditions caused by exposure to lethal or permanently disabling toxic biological, chemical, radiological, or nuclear substances. This subpart applies only to those new drug products for which definitive human efficacy studies cannot be conducted because it would be unethical to deliberately expose healthy human volunteers to a lethal or permanently disabling toxic biological, chemical, radiological, or nuclear substance; field trials to study the product's effectiveness after an accidental or hostile exposure have not been feasible.

(ii) Sec. 314.610 Approval based on evidence of effectiveness from studies in animals.

(a) FDA may grant marketing approval for a new drug product for which safety has been established and for which the requirements of 314.600 are met based on adequate and well-controlled animal studies when the results of those animal studies establish that the drug product is reasonably likely to produce clinical benefit in humans. In assessing the sufficiency of animal data, the agency may take into account other data, including human data, available to the agency. FDA will rely on the evidence from studies in animals to provide substantial evidence of the effectiveness of these products only when

1. there is a reasonably well-understood pathophysiological mechanism of the toxicity of the substance and its prevention or substantial reduction by the product;
2. the effect is demonstrated in more than one animal species expected to react with a response predictive for humans, unless the effect is demonstrated in a single animal species that represents a sufficiently well-characterized animal model for predicting the response in humans;
3. the animal study endpoint is clearly related to the desired benefit in humans, generally the enhancement of survival or prevention of major morbidity;
4. the data or information on the kinetics and pharmacodynamics of the product or other relevant data or information, in animals and humans, allows selection of an effective dose in humans.

To date, nine years after establishing the Animal Rule, no biologic product has been licensed using this regulatory pathway [18]. The two approvals under the Animal Rule were for new indications for drugs that had previously been approved for other indications. An analysis in 2008 of the costs and likelihood of success for medical countermeasures estimated the failure rate at more than 85% [19].

FDA's budget request for FY 2012 includes the following for medical countermeasures [18]:

FDA MCM Objective (1)—Enhance the Review Process for MCM by Establishing Public Health and Security Action Teams (PHSATs) (+$24,199,000/85 FTE);
FDA MCM Objective (2)—Advance Regulatory Science for MCM Development and Evaluation (+$36,903,000/60 FTE);
FDA MCM Objective (3)—Modernize the Legal, Regulatory, and Policy Framework for Effective Public Health Response (+$5,267,000/20 FTE).

These requests for funding to enhance the review process and advance the regulatory science for MCM at FDA suggest that an ill-prepared FDA has somehow been responsible for the slow process of bringing MCM to approval or licensure, rather than that the chemistry, manufacturing and quality control of MCM, and the efficacy requirements of the Animal Rule are themselves inherently difficult.

### 4.2. FDA Background on Anthrax

FDA has been proactive about the development of vaccines and therapeutics for anthrax since the attacks of 2001:

- April 2002: FDA, the National Institute for Allergy and Infectious Diseases, and the Department of Defense sponsored a workshop on Anthrax Vaccines: Efficacy Testing and Surrogate Markers of Immunity Workshop;
- June 2004: FDA, the Centers for Disease Control, and the National Institutes of Health sponsored a workshop on Strategies for Developing Therapeutics that Directly Target Anthrax and its Toxins;
- November 2007: FDA, the National Institute of Allergy and Infectious Diseases, and the Department of Health and Human Services Office of Biomedical Advanced Research and Development Authority sponsored a workshop on Anthrax Vaccines: Bridging Correlates Of Protection In Animals To Immunogenicity In Humans;
- November 2010: FDA's Vaccines and Related Biological Products Advisory Committee considered the Pathway to Licensure for Protective Antigen-based Anthrax Vaccines for a Postexposure Prophylaxis Indication Using the Animal Rule.

The 2004 workshop on therapeutics included sessions on

- pathogenesis of *B. anthracis*;
- in vitro characterization;
- animal studies;
- human testing; and
- challenges and opportunities in product development.

The session on animal studies included the following.

- The Animal Rule applied Pyridostigmine for Nerve Gas Exposure and Gentamicin for Plague.
- GLP issues.
- Animal efficacy:
  - species: mouse, rat, hamster, guinea pig, rabbit, and nonhuman primate (cynomolgus rhesus),
  - technical methods for animal studies;
- Panel discussion.

FDA did not present its current thinking on animal efficacy studies in a prepared talk but rather addressed questions to the panel. The FDA moderator recognized that antibiotic therapies would likely be used in conjunction with antibodies and perhaps other classes of agents; therefore, he asked the panel members how they envisioned designing efficacy studies when more than one agent was being studied, in order to identify the correct timing and dosing of the investigational therapeutic for (a) the postexposure prophylaxis indication and (b) the treatment indication. 

The first point made was that each agent needs to be understood individually, before studying combinations, in order to have a better idea about timing for the combination protocol. The moderator responded by asking if it would be sufficient to study each agent individually, to show that each is better than a control, and the reply to that was it will depend on what the product is expected to do. An FDA representative on the panel noted that it could be a complicated and resource-intensive matter to determine whether an agent is synergistic or additive when used in combination. An FDA representative not on the panel agreed that in the more complicated case of treatment, as opposed to prophylaxis, particularly if there is the potential of using the agent in combination rather than individually, the developer would need to talk with the review division before starting to design the studies. He said that FDA was looking forward to interacting with those who are developing interventions to try to make the process as efficient as possible.

With regard to timing of treatment in a therapeutic study, an audience member asked if it would be reasonable to treat cohorts of animals at successively later times, rather than monitoring animals around the clock for fever. An FDA representative on the panel replied that it depends on what the study is trying to prove**—**if the developer wants to say that an intervention should be used upon evidence of fever, they would have to monitor their animals and design the experiment contingent upon fever, because the instructions for use would be based on evidence of fever. Another audience member noted that he had heard nothing about the actual clinical manifestations occurring in animals, nothing to help him design studies in animals that would help to treat symptomatic anthrax. He noted that for some of the models that had been discussed, after the animals are infected with the agent, the next thing that is clinically apparent is death, which is a little late to treat. He wanted to hear about data from animal models that could be used for designing studies for the treatment of symptomatic anthrax infection. (Applause noted in the transcript at this point.) An FDA representative agreed that there was a lot of information that was just not there.

Perhaps the most salient comment in all these public venues with respect to the Animal Rule was at the 2007 workshop in the form of the following quotation from statistician George Box.

Essentially, all models are wrong, but some are useful [20].

## 5. Approvals under the Animal Rule

### 5.1. Pyridostigmine Bromide (PB)

PB was approved in the United States in 1955 for the treatment of myasthenia gravis, a rare neurological disorder of too few acetylcholine (ACh) receptors resulting in muscle weakness. Acetylcholinesterase (AChE) is widely present in the body and breaks down ACh. PB is a reversible inhibitor of AChE and thus allows ACh to remain present longer, resulting in improved muscle strength. Too much PB, however, can result in too much ACh, leading to chronically stimulated muscles that quickly result in paralysis.

Soman is an irreversible inhibitor of AChE and can similarly result in paralysis. When Soman initially binds to AChE, it can be displaced by pralidoxime, but within minutes the binding becomes irreversible. The benefit of PB in *pretreatment *for Soman poisoning stems from its reversible binding to AChE, protecting some of the AChE from inactivation by Soman. Pralidoxime and atropine must be administered within minutes of Soman exposure. When a PB molecule leaves an AChE molecule in the free state, any residual Soman that might initially bind to the free AChE can be displaced by pralidoxime.

On 3 January 2003, barely seven months after the final Animal Rule was issued, the Army resubmitted its NDA for PB to establish that the elements of the rule had been met. On 5 February 2003, FDA approved the application with indications and usage as follows.

Pyridostigmine bromide is indicated for prophylaxis against the lethal effects of Soman nerve agent poisoning. Pyridostigmine is intended to be used in conjunction with protective garments, including a gas mask and immediate atropine and pralidoximine therapy at the first sign of nerve agent poisoning. Pyridostigmine should be stopped at the first sign of nerve agent poisoning.

The evidence for the effectiveness of pyridostigmine as prophylaxis against Soman-induced toxicity was derived from animal studies alone.

There was nothing slow about FDA's review and approval of the Army's application under the new Animal Rule. Dr. Temple's memorandum supporting approval noted that there had been discussion and, to a degree, disagreement about whether the expectations of the rule had been met and the relevance of those data to humans.

Dr. Temple noted that there are good explanations, supported by considerable data, carried out by many investigators, of the apparent differences between animal species in their protective ratio to PB (and their different sensitivities to Soman) that mitigate the concern arising from the apparent minimal protection in some species. The difference in protective ratios between monkeys/guinea pigs and rodents/rabbits can be explained by different levels of a Soman-binding enzyme, carboxylesterase, in different species that protects these species (rodents in particular) from Soman poisoning. Based on human carboxylesterase levels, the response of humans to PB is thus far more likely to be similar to that seen in monkeys and guinea pigs (a substantial protection for Soman lethality) than to the smaller protective ratio effect in rabbits and rodents. 

Dr. Temple noted that the data strongly support the proposed mechanism of action of PB in protecting against Soman toxicity (requirement 1) and lead to a conclusion that the results in monkeys and guinea pigs will be predictive of the results in humans (requirement 2). He thus concluded that we do understand “reasonably well” the pathophysiology of the protective effect of PB and that the effect seen in monkeys and guinea pigs is in fact expected to be predictive of an effect in humans [21].

### 5.2. Cyanokit

Cyanokit is a lyophilized formulation of hydroxocobalamin for use as an antidote in treating patients with known or suspected cyanide poisoning. Cyanide displaces the hydroxyl group in hydroxocobalamin, resulting in the formation of cyanocobalamin, which is vitamin B12. Hydroxocobalamin has been an approved drug for decades for treatment of vitamin B12 deficiency, albeit at thousands of fold lower doses than used in Cyanokit. The currently marketed generic form of hydroxocobalamin was approved in 1978.

Cyanokit was granted marketing authorization in France in May 1996 based on one prospective study and several retrospective studies in victims of smoke inhalation that included blood sampling prior to hydroxocobalamin treatment for measurement of cyanide levels, along with one retrospective study in subjects who had been exposed to cyanide by other than fire or smoke. None of the studies were placebo controlled, as expected when testing an antidote anticipated by its mechanism of action to be effective. The study results indicated that many of the treated subjects survived what would otherwise have been lethal doses of cyanide. FDA considered that the lack of a comparator limited the interpretation of these findings, concluding only that the levels of cyanide in humans in the French studies were similar to cyanide levels that are lethal in dogs. Survival in the French studies was very low for subjects who presented in cardiac arrest, but quite high for subjects who were not in cardiac arrest. 

FDA requested that Merck Santé consider seeking approval of Cyanokit in the United States. In March 2001, FDA met with Orphan Medical, Inc., which had entered into a letter of intent with the Merck subsidiary. FDA's medical review of Cyanokit describes that the requirements for an NDA were established at that meeting and several following interactions and included efficacy studies in animals conducted in accordance with 21 CFR 314 Subpart I. Orphan Medical undertook the animal studies and submitted an NDA with the expected results demonstrating efficacy. Cyanokit was approved in the United States in December 2006, based primarily on a single placebo-controlled study in a single species (dogs) [22].

## 6. An Almost-Approved Anthrax Antidote

### 6.1. Commercial Development of an Anthrax Antidote

With regard to developing medical countermeasures for treatment of anthrax disease, Human Genome Sciences (HGS) announced the following in press releases or in the briefing package for the October 2009 meeting of the Vaccines and Related Biological Products Advisory Committee [23, 24].

- October 2002: pre-IND meeting between HGS and FDA;
- February 2003: use of protein and antibody drug development capabilities to develop therapeutic candidates to address microbial targets including anthrax;
- March 2003: discovery of a human monoclonal antibody drug that is effective in protecting against anthrax in multiple experimental models in animals;
- May 2003: submitted IND to FDA;
- June 2003: clearance from the Food and Drug Administration (FDA) to begin human trials of raxibacumab;
- July 2003: initiation of clinical development of raxibacumab for the prevention and treatment of anthrax infections;
- August 2003: designation from the FDA for raxibacumab as a Fast Track Product;
- November: orphan drug designation for raxibacumab granted by FDA;
- March 2004: finding in the Phase 1 trial that raxibacumab is safe and well tolerated in healthy volunteers and achieved the blood levels predicted by relevant animal models as necessary to afford significant protection from the lethal effects of the anthrax toxin;
- 2004 to 2007: a series of meetings with FDA regarding the additional animal efficacy studies needed to support licensure and/or use in the Strategic National Stockpile. These interactions established;
  - the requirement to demonstrate efficacy in two species (rabbits and monkeys);
  - that the animals had to have evidence of systemic anthrax disease at the time of raxibacumab administration for an indication in therapeutic treatment;
  - that serum protective antigen (PA) could be used as a trigger for therapeutic treatment;
  - that the antibiotic exposure in animals in the raxibacumab/antibiotic combination studies should approximate the exposure achieved by the recommended dose in humans.

Agreement on the division of studies between those needed to support submission of an IND by the CDC to use raxibacumab in the Strategic National Stockpile and those additional studies needed for licensure was also achieved. During 2007, HGS submitted the protocols and analysis plans for the rabbit and monkey efficacy studies. The protocols for the rabbit and monkey raxibacumab/antibiotic combination studies were completed in the summer of 2008.

- July 2005: publication of Phase 1 study results in Clinical Infectious Diseases;
- October 2005: award for a two-phase contract to supply raxibacumab to the United States Government. Under the first phase of the contract, HGS would supply ten grams of raxibacumab to the Department of Health and Human Services for comparative *in vitro* and *in vivo* testing. Under the second phase of the contract, the Government had the option to order up to 100,000 doses of raxibacumab for the Strategic National Stockpile;
- June 2006: government exercised option to purchase 20,000 treatment courses of raxibacumab for the Strategic National Stockpile;
- October 2008: Pre-Biologics License Application meeting with FDA;
- February 2009: commencement of delivery of 20,000 doses raxibacumab to the Strategic National Stockpile;
- May 2009: submission of a Biologics License Application (BLA) for raxibacumab;
- July 2009: publication in The New England Journal of Medicine of the results of two pivotal animal efficacy studies, which showed the life-saving potential of raxibacumab in the event of life-threatening inhalation anthrax disease;
- July 22, 2009: Government exercised option to purchase additional 45,000 doses of raxibacumab for the Strategic National Stockpile;
- October 2009: consideration of the BLA for raxibacumab at FDA's Anti-Infective Drugs Advisory Committee;
- November 2009: FDA issued Complete Response Letter requesting additional information relating to the BLA for raxibacumab;
- March 2010: $180.2 million in sales of raxibacumab to the Strategic National Stockpile in 2009 (the first product sales for the company);
- January 2011: HGS working closely with FDA to obtain approval of raxibacumab for the treatment of inhalation;
- February 2011: $47.2 million in sales of raxibacumab to the Strategic National Stockpile in 2010.

### 6.2. FDA Advisory Committee

In October 2009, FDA's Anti-Infective Drugs Advisory Committee convened to consider the Biologics License Application from Human Genome Sciences for raxibacumab injection, a monoclonal antibody product for treatment of inhalation anthrax [25]. At the outset of the meeting, FDA indicated that results of inspections of bioanalytical assays for raxibacumab and ciprofloxacin raised questions about the reliability of the clinical pharmacology data. Therefore, FDA would discuss neither pharmacokinetic or pharmacodynamic results nor the selection of a human dose.

### 6.3. Human Genome Sciences' Perspective

Dr. Sally Bolmer of Human Genome Sciences presented results of anthrax challenge studies in New Zealand white rabbits and cynomolgus monkeys, both well-characterized models of inhalational anthrax. Raxibacumab doses of 20 and 40 mg/kg achieved concentrations that were at least as high as the highest PA levels in anthrax-infected animals. The pivotal animal efficacy studies had parallel groups, with animals randomized to raxibacumab or placebo. To mimic the human clinical situation, other studies were conducted in which animals in some of the treatment groups received antibiotics at the onset of symptoms after anthrax challenge. Dr. Bolmer noted, however, that the preamble to the Animal Rule describes the need for a wide range of therapeutic options for the treatment of bioterror pathogens and specifically cites anthrax. She noted further that the preamble states that there is no requirement for new therapies to provide meaningful therapeutic benefits to subjects over existing therapies, nor does it require the toxic agent to be without a proven treatment. Finally she reported that concomitant administration of raxibacumab with antibiotics in animals did not alter the antibiotic efficacy or pharmacokinetics. In other words, raxibacumab was effective in the animal models, and antibiotics were effective in the animal models, and there was no decrease in effectiveness when raxibacumab was administered with antibiotics. Dr. Bolmer concluded her remarks by introducing Dr. Daniel Lucey, who was chief of the Infectious Disease Service at the Washington Hospital Center in Washington, DC, during the anthrax attacks in 2001.

Dr. Lucey noted that in controlled experimental settings, antibiotics can achieve up to 100% survival but that in real world clinical use antibiotics are not as successful. He noted that in the United States in the 20th century, mortality from inhalational anthrax was 90% with antibiotic susceptible strains of anthrax [26]. In the anthrax attacks in 2001, survival was 55% in the 11 patients with inhalational anthrax, and Dr. Lucey considered that more rapid blood culture results, use of two or more antibiotics (ideally at least one which crosses the blood-brain barrier in order to prevent or treat anthrax meningitis), and pleural fluid drainage have improved survival. Nevertheless, the mortality rate of 45% was highly unacceptable, and he considered that an antitoxin is needed as an additional treatment modality, because the toxins still exert deleterious effects after control of bacterial replication with antibiotics. 

Dr. Lucey went on to say that prior to the development of symptoms, antibiotics were very effective for postexposure prophylaxis in 2001. Even in the first one to five days of prodromal flu-like symptoms, prompt treatment with antibiotics may have great benefit. In an inhalational anthrax infection, the prodromal phase is followed by an intermediate progressive phase characterized by bacteremia and/or pleural effusions and/or mediastinal adenopathy. Lastly, progression to the late fulminant stage can occur rapidly and lead to death within 6 to 24 hours. At this late fulminant stage, there has been no demonstrated benefit from antibiotics alone, and all five patients in 2001 who died had reached this stage. Finally, Dr. Lucey noted that the treatment paradigm of using an antitoxin in combination with antimicrobials is well established for tetanus.

 Dr. Thi-Sau Mignone of Human Genome Sciences described the pivotal animal studies with raxibacumab in detail. The challenge dose of anthrax was 200 times the LD_50_ (the dose that causes lethality in 50% of the animals). At this dose, the majority of rabbits died between days 3 and 5 whereas the majority of monkeys died between days 4 and 6. Positive bacteremia and anthrax PA in blood occurred at 24 hours in the rabbits and at 36 hours in the monkeys. A significant rise in temperature was also seen in rabbits at 24 hours, but monkeys have a strong diurnal rhythm, and temperature rise was not observed at 36 hours. When raxibacumab was administered at 40 mg/kg at the onset of these symptoms, 44% of the rabbits and 64% of the monkeys survived.

The studies in which antibiotics and raxibacumab were coadministered upon onset of symptoms, as would be expected in clinical practice in humans, were conducted to test whether raxibacumab would interfere with levofloxacin or ciprofloxacin. The studies were not designed to detect superiority or noninferiority of the combined treatment to treatment with the antibiotic alone but rather to detect superiority of the combined treatment to placebo. In both rabbits and monkeys, survival was statistically significant higher with combined raxibacumab and levofloxacin (rabbits) or ciprofloxacin (monkeys) than with placebo, but survival was not different between combined treatment and treatment with the antibiotic alone.

In contrast to the animal models, in humans in the anthrax attacks of 2001, symptoms developed 4 to 6 days after exposure, and death occurred 5 to 8 days after the appearance of symptoms, for a total time to death in the range of 9 to 14 days. 

Dr. Dan Hanfling, special advisor for emergency preparedness and response for the Inova Health System and clinical professor of emergency medicine at George Washington University, reviewed the risk-benefit profile of raxibacumab. He had been integrally involved in the emergency management response during the 2001 attacks in the national Capitol region, during which time two postal workers were successfully diagnosed and treated. He noted that over 33,000 people were treated with prophylactic antibiotics after the 2001 attack and reported that the Commission on the Prevention of Weapons of Mass Destruction had recently warned that anthrax spores released by a crop duster could kill more Americans than died during all of World War II. Although Human Genome Sciences had not tested antibiotic-resistant strains of anthrax, he pointed out that PA is highly conserved in all known strains of anthrax and that raxibacumab would thus as a single agent be expected to be effective against antibiotic-resistant strains. He concluded that it is reasonable to predict that the benefit to risk profile in humans is strongly positive for raxibacumab as a treatment for anthrax infection. 

### 6.4. FDA's Perspective

Dr. Yuliya Yasinskaya, a medical officer in the Division of Special Pathogens and Transplant Products at the Center for Drug Evaluation and Research, presented an FDA perspective on the raxibacumab animal studies. She pointed out that the animal studies were subject to several limitations in predicting response in humans, among which is the time of intervention, which could be different between animals in a controlled research environment and humans in a clinical setting. Antibiotics were very effective in the animal studies, with close to 100% survival. 

FDA was also quite concerned about the unexpected finding of a greater incidence and severity of central nervous system (CNS) lesions in raxibacumab-treated animals that died compared to placebo animals that died. Dr. Yasinskaya noted that although only a single rabbit and a single monkey died in the combined treatment groups, there were high grade CNS lesions in 100% of those animals, whereas in the placebo group the incidence of high grade lesions was low. Dr. Yasinskaya concluded that the question remains whether in humans the addition of raxibacumab added to antibiotics will provide either additional benefit or additional risk. Due to limitations of the animal studies, it remained uncertain how long antibiotic treatment can be delayed and thus how late raxibacumab might be able to provide improved survival in these models.

### 6.5. Advisory Committee's Perspectives

After much preliminary discussion, the FDA questions were read and the advisory committee voted as follows.

1. Does the evidence from the animal models evaluating raxibacumab at 40 mg/kg IV predict response for treatment of humans with inhalational anthrax disease? And if not, what additional studies should be conducted?

One committee member interpreted the question to refer to monotherapy. Another committee member noted that the question could be taken to mean response at any time postexposure or at any stage of clinical illness.

The committee voted 16 in favor, 7 opposed, with 1 abstention. Several of the committee members, whether they voted in favor or opposed, expressed a desire to see data from animal studies in which antibiotic treatment was not 100% successful, either because doses would be more in keeping with human doses or because timing of administration would be delayed, so that additional benefit of raxibacumab could be observed if additional benefit indeed exists. It was noted that in a disaster situation, humans would probably show up a week or more after their exposure, after they probably have been PA positive for a while, a very different situation than the animal studies. 

One committee member remarked that the wording of the question was almost Talmudic, and the people who voted in favor or opposed often had all the same reasons and all the same concerns but wound upcoming down on one side or the other of the fence. One committee member who voted in favor commented she thought elements 1 through 3 of the Animal Rule were met and that we should move forward with this kind of drug. Several other committee members who voted in favor stated that they narrowly interpreted the question as referring to monotherapy with raxibacumab. One committee member explained his opposition as due to the wording of the question with respect to anthrax disease, pointing out that appearance of PA in the blood of animals is not anthrax disease.

(2) Does the evidence provided in these studies support the conclusion that raxibacumab will not diminish the anticipated efficacy of antimicrobials in inhalational anthrax? And if not, what additional studies should be conducted?

One committee member immediately noted that FDA had instructed the committee to ignore the pharmacokinetic data, because of questions about their reliability. Therefore, he asked how is the committee to answer this question if ignoring the pharmacokinetic data? FDA suggested that he should vote as he believed he could vote and then provide his perspective as to why he took that vote and how he might have voted depending on what kind of clinical data were available.

The committee voted 10 in favor, 11 opposed, with 3 abstentions. The first committee member called upon to explain his vote noted that there was a major control arm missing, raxibacumab alone, so that it was very difficult for him to make that comparison. Another who voted in opposition said that the antibiotics had been given in doses so far above their therapeutic thresholds that if there was a detrimental effect of raxibacumab, it might not have been seen in these studies. One committee member voted in favor because he knew of no precedent for a protein therapeutic like an antibody to alter the behavior of a small molecule antimicrobial. Another who voted in favor said that there was no evidence to the contrary. 

The statistician committee member calculated that it would take about 400 animals to detect a statistically significant difference between 95% survival in one group versus 85% in the other. He considered that a study with that number of animals sounded like a nonstarter. One member said he abstained because he could not decide which way to vote and that the committee vote had validated his feelings. Eventually, one member said that he voted in opposition because of the narrow construction of what was presented and that he voted in favor (laughter noted in the transcript at this point) because of the narrow construction that the percentages were the same. Finally one member said that the last time he looked in a biochemistry textbook, antibiotics can be protein bound and that there was a serious matter here and that the animals should be treated with antibiotics at doses that we would use to treat a human being.

(3) Should evidence be requested that raxibacumab makes a contribution to the efficacy over the antimicrobial alone in rabbit and monkey models? If yes, what types of additional studies should be requested and then conducted?

The committee voted 17 in favor, 6 opposed, with 1 abstention. A common sentiment was that in additional studies, the animals should be given the study material at a time that is truly therapeutic and not prophylactic. One member who voted in favor noted that the question did not say who would conduct the studies and he was not sure that the “poor sponsor” who had the misfortune of developing this compound should have to do all those studies. Another member who voted in favor thought that it might be a difficult bar for the sponsor to meet. A member who voted in opposition pointed out that raxibacumab may have benefit as monotherapy if it is dosed correctly, although it would be good to know if it could help rescue patients that had delayed antibiotic therapy. Another member who voted in opposition said it may be an unreasonable burden to say raxibacumab is going to benefit every patient who receives it. He thought there was every reason to think raxibacumab is safe and efficacious and would benefit a significant fraction of those who receive it.

(4) Do you have any recommendations how this (the CNS findings) might be further evaluated?

Despite a number of committee members advocating further studies in a meningitis model, one member pointed out that their understanding was biased by the fact that the only data were from nonsurviving animals. He noted that the only way to determine whether there was causality would be to look at survivors as well to see if they have any CNS toxicity. A couple of committee members were concerned about antibody complexes as potentially a source of toxicity.

(5) Are there additional comments or further recommendations for safety evaluation in humans? If yes, what are these recommendations?

One committee member recommended that some work needs to be done to make sure that raxibacumab would be safe for use in children, and another recommended that the same level of assurance was needed for elderly patients.

## 7. Discussion

### 7.1. Pyridostigmine Bromide (PB)

In 2008, the congressionally appointed Research Advisory Committee on Gulf War Veterans' Illnesses reported that roughly 25% of the 697,000 veterans were afflicted. Exposure to PB and pesticides that were heavily used during the war was implicated as contributing to the illnesses [27].

A National Academy of Sciences committee, however, reviewed the evidence in the Research Advisory Committee's report and published in 2010 that human epidemiologic evidence was not sufficient to establish a causative relationship between any specific drug, toxin, plume, or other agent, either alone or in combination, and Gulf War illness [28].

The Animal Rule was born of the controversy over administration of investigational drugs and biologics to soldiers under military orders without informed consent, under an administrative rule for waiver of informed consent in certain military exigencies. Now that PB has been licensed by FDA under the Animal Rule, should there be future military exigencies in which the use of Soman is anticipated, informed consent would no longer be an issue in ordering soldiers to take the licensed drug as a pretreatment against possible attack. Thus the legal situation for use of PB has changed, but the safety profile of PB has of course not changed at all because of its regulatory transition from an investigational to a licensed drug.

### 7.2. Cyanokit

FDA considered the approval of Cyanokit to have been based primarily on the dog study, despite the fact that one prospective and several retrospective studies had been conducted in humans. Apparently FDA was more willing to extrapolate efficacy from dogs to humans than it was to make a medical decision about the efficacy of Cyanokit based on the open label studies in France. FDA appeared reluctant to stray from its gold standard of randomized, blinded, and placebo-controlled trials, apparently giving more credence to statistical analyses of data from dogs than to medical evidence from humans.

FDA could have compared the cyanide levels in the French patients who were in cardiac arrest and died to the cyanide levels in patients who were not in cardiac arrest at the time of treatment and lived. It would not have been difficult to evaluate whether a number of patients not in cardiac arrest had baseline cyanide levels that were fatal in subjects who had progressed to cardiac arrest before treatment, but FDA would have had to have wanted to see what could be learned from the French data. As noted above, FDA concluded only that cyanide levels in the French patients were similar to cyanide levels that are lethal in dogs and therefore was apparently unwilling to make a medical decision based on evidence that did not come with a gold star of statistical significance. In the end, more than five years after Merck Santé was solicited to seek approval and conducted a randomized, placebo-controlled animal study, FDA approved Cyanokit as effective, as the French regulatory authority had already done on the basis noncontrolled human studies.

### 7.3. Raxibacumab

Regarding the specific matter of CNS toxicity, since raxibacumab does not cross an intact blood-brain barrier, the basis for FDA's concern is not completely clear. After all, if raxibacumab is effective in other tissues in preventing damage from anthrax toxins, one might expect delayed progression of disease in those tissues—and indeed time to death was longer in the raxibacumab-treated animals that died compared to placebo-treated animals that died—thus leaving more time for lesions to develop in the brain. In fact, in raxibacumab-treated rabbits in the pivotal study, the incidence and severity of histopathology in nonsurvivors were the same or lower than in placebo-treated rabbits for all tissues except for the brain. In the lungs in particular, the pathology was severe in the placebo-treated animals but lower in the raxibacumab-treated animals. In the words of Dr. Mignone, the CNS lesions were more likely due to a difference in the site of benefit for raxibacumab than to an actual deleterious effect. Even if the blood-brain barrier is compromised as anthrax disease progresses and raxibacumab then does enter the CNS, it is not clear why some advisory committee members were concerned about a monoclonal antibody forming immune complexes with the PA protein, since raxibacumab binds only to a single epitope on PA and would not be expected to cross-link the PA proteins in brain. 

Regarding the specific matter of antibiotic dosing in the combination studies, the HGS briefing package notes that 50 mg/kg in the rabbit produces similar exposure to that reported for the approved human doses of 500 or 750 mg levofloxacin. Since many humans weigh between 50 kg to 75 kg, the human doses are thus in the range of 10 mg/kg. HGS was unable to rebut the advisory committee's criticism that they had used doses in the animals that were in excess of the human doses, because FDA had taken any discussion of the pharmacokinetic data off the table. Taking the briefing package statement about similar antibiotic exposure at face value and recognizing that HGS and FDA had concurred that the antibiotic exposure in animals should approximate that of the recommended dose for humans, the advisory committee's criticism appears to have been misplaced. 

Regarding the general matter of FDA providing guidance to HGS during the entire course of clinical development and specifically on the protocols for the animal studies, it appears to be an awkward regulatory posture for FDA to have prospectively concurred with the design of the combination raxibacumab-antibiotic studies but then to have retrospectively required that HGS needed to evaluate whether or not raxibacumab provides additional benefit beyond that provided by antibiotics alone. The question as to whether additional benefit had to be demonstrated for use of a new therapeutic intervention in combination with antibiotics had been raised during the workshop in 2004, so FDA was very well aware of the matter. Further, HGS had conducted a pilot study of levofloxacin in rabbits challenged with anthrax, with levofloxacin dosing triggered by rise in temperature. Levofloxacin was quite effective at 10 mg/kg, 25 mg/kg, and 50 mg/kg, the latter of which in rabbits was found to be equivalent in exposure to the approved human dosing. The results of this study had been submitted to FDA and had predicted that survival would be very high in the proposed combination study of levofloxacin and raxibacumab, meaning that an additional benefit of raxibacumab could not be detected, only as a detriment. One can only assume from the outside that the FDA review team decided that a showing of additional benefit for raxibacumab beyond the benefit of antibiotics alone was not necessary, at the same time that they reviewed the HGS protocol designed to evaluate whether raxibacumab had a detrimental effect on antibiotic efficacy. The FDA recommendation at the 2004 workshop for a developer to talk with the FDA review division before commencing animal studies had years later appeared in the January 2009 Draft Guidance for Industry: Animal Models**—**Essential Elements to Address Efficacy Under the Animal Rule.

FDA strongly encourages sponsors to submit a development plan and to communicate frequently with the agency when developing products under the Animal Rule. The protocols for the animal efficacy studies should be discussed with FDA, with sufficient time for FDA review and comment, prior to the study being conducted.

From HGS's description of its interactions with FDA from 2004 to 2007 on the protocols for animal efficacy studies, it would appear that HGS comported with what is now FDA guidance. On FDA's side, after the workshop in 2004 on the very topic of Strategies for Developing Therapeutics that Directly Target Anthrax and its Toxins, the review team should have been better prepared for providing guidance to HGS for development of raxibacumab than they appear to have been.

## 8. Conclusions

In its FY 2012 budget request, FDA requested $36.9 million for advancing regulatory science for MCM development and evaluation, which amounts to funding for 60 full-time equivalents. However, it was not lack of FDA personnel that led to nonlicensure of raxibacumab, but rather lack of forethought by FDA personnel as to whether HGS needed to demonstrate that their antidote to anthrax toxin provides additional benefit to antibiotics for treatment of inhalational anthrax disease. 

Tens of thousands of vials of raxibacumab now reside in the Strategic National Stockpile, as investigational drugs, and no doubt would be used in the event of future cases of patients with advanced inhalational anthrax disease. Clearly there have been different standards for national stockpiling on one hand and for FDA approval of drugs or licensure of biologics on the other as medical countermeasures for biothreats. At least two other investigational biologic drugs, Anthrax Immune Globulin and Botulism Antitoxin Heptavalent, also reside in the Strategic National Stockpile [29].

The Animal Rule was developed to provide a basis for approving certain drugs or licensing certain biologic products as medical countermeasures to biological threats without efficacy data in humans, the alternative being to leave these products languishing as investigational drugs that require informed consent from human subjects prior to use. Personally and for the sake of his children, the author is relieved that promising medical countermeasures do not need FDA approval or licensure in order to be produced in large quantities and stockpiled for ready availability in the event of a mass bioterror attack on the United States.

## References

[1] Franco C (2009). Billions for biodefense: federal agency biodefense funding, FY2009-FY2010. *Biosecurity and Bioterrorism*.

[2] HHS Public Health Emergency Medical Countermeasure Enterprise Implementation Plan for Chemical, Biological, Radiological and Nuclear Threats. https://www.medicalcountermeasures.gov/BARDA/documents/phemce_implplan_041607final.pdf.

[3] Robinson JP, Goldblat J Chemical Warfare in The Iran-Iraq War 1980 1988. http://www.iranchamber.com/history/articles/chemical_warfare_iran_iraq_war.php.

[4] http://www.un.org/Depts/unscom/General/basicfacts.html.

[5] Cole LA (2003). *The Anthrax Letters: A Medical Detective Story*.

[6] Powell C Remarks to the United Nations Security Council. http://www.guardian.co.uk/world/2003/feb/05/iraq.usa.

[7] Mowatt-Larssen R (2010). Al Qaeda’s Pursuit of Weapons of Mass Destruction. *Foreign Policy*.

[8] Report to the President of the United States (March 2005). U.S. Commission on the Intelligence Capabilities of the United States Regarding Weapons of Mass Destruction.

[9] Rettig R Military Use of Drugs Not Yet Approved by the FDA for CW/BW Defense: Lessons from the Gulf War. http://www.rand.org/pubs/monograph_reports/MR1018.9/index.html.

[13] Administrative documents on FDA website under Drugs@FDA. http://www.accessdata.fda.gov/scripts/cder/drugsatfda/index.cfm?fuseaction=Search.DrugDetails.

[18] FDA FY 2012 Budget Request. http://www.fda.gov/downloads/AboutFDA/ReportsManualsForms/Reports/BudgetReports/UCM243370.pdf.

[19] Matheny J, Mair M, Smith B (2008). Cost/success projections for US biodefense countermeasure development. *Nature Biotechnology*.

[20] Box G, Draper R (1987). *Empirical Model-Building and Response Surfaces*.

[21] Administrative documents on FDA website under Drugs@FDA. http://www.accessdata.fda.gov/scripts/cder/drugsatfda/index.cfm?fuseaction=Search.DrugDetails.

[22] Administrative documents on FDA website under Drugs@FDA. http://www.accessdata.fda.gov/drugsatfda_docs/nda/2006/022041_cyanokit_toc.cfm.

[23] http://www.hgsi.com/.

[24] http://www.fda.gov/downloads/AdvisoryCommittees/CommitteesMeetingMaterials/Drugs/Anti-InfectiveDrugsAdvisoryCommittee/UCM187312.pdf.

[25] http://www.fda.gov/downloads/AdvisoryCommittees/CommitteesMeetingMaterials/Drugs/Anti-InfectiveDrugsAdvisoryCommittee/UCM191757.pdf.

[26] Holty JEC, Bravata DM, Liu H, Olshen RA, McDonald KM, Owens DK (2006). Systematic review: a century of inhalational anthrax cases from 1900 to 2005. *Annals of Internal Medicine*.

[27] Gulf War Illness and the Health of Gulf War Veterans, Research Advisory Committee on Gulf War Veterans’ Illnesses. http://www.va.gov/RAC-GWVI/Committee_Documents.asp.

[28] (2010). Gulf War and Health: Volume 8: Update of Health Effects of Serving in the Gulf War. http://www.nap.edu/catalog.php?record_id=12835#orgs.

[29] http://cangene.com/biodefense_products.shtml#aig.

